# An On-Site Simultaneous Semi-Quantification of Aflatoxin B1, Zearalenone, and T-2 Toxin in Maize- and Cereal-Based Feed via Multicolor Immunochromatographic Assay

**DOI:** 10.3390/toxins10020087

**Published:** 2018-02-17

**Authors:** Lin Xu, Zhaowei Zhang, Qi Zhang, Wen Zhang, Li Yu, Du Wang, Hui Li, Peiwu Li

**Affiliations:** 1 Oil Crops Research Institute of the Chinese Academy of Agricultural Sciences, Wuhan 430062, China; xulinlin2008@126.com (L.X.); zhangwen@oilcrops.cn (W.Z.); yuli0201010133@hotmail.com (L.Y.); wang416929@126.com (D.W.); lihui-gf@163.com (H.L.); 2 Key Laboratory of Biology and Genetic Improvement of Oil Crops, Ministry of Agriculture, Wuhan 430062, China; 3 Key Laboratory of Detection for Mycotoxins, Ministry of Agriculture, Wuhan 430062, China; 4 Laboratory of Risk Assessment for Oilseeds Products (Wuhan), Ministry of Agriculture, Wuhan 430062, China

**Keywords:** multicolor, immunochromatographic assay, aflatoxin B1, zearalenone, T-2

## Abstract

Multiple-mycotoxin contamination has been frequently found in the agro-food monitoring due to the coexistence of fungi. However, many determination methods focused on a single mycotoxin, highlighting the demand for on-site determination of multiple mycotoxins in a single run. We develop a multicolor-based immunochromatographic strip (ICS) for simultaneous determination of aflatoxin B1 (AFB1), zearalenone (ZEN) and T-2 toxin in maize- and cereal-based animal feeds. The nanoparticles with different colors are conjugated with three monoclonal antibodies, which serve as the immunoassay probes. The decrease in color intensity is observed by the naked eyes, providing simultaneous quantification of three mycotoxins. The visible limits of detection for AFB1, ZEN and T-2 are estimated to be 0.5, 2, and 30 ng/mL, respectively. The cut-off values are 1, 10, and 50 ng/mL, respectively. Considerable specificity and stability are found using real samples. The results are in excellent agreement with those from high-performance liquid chromatography/tandem mass spectrometry. The multi-color ICS boasts sensitive and rapid visual differentiation and simultaneous semi-quantification of aflatoxin B1, zearalenone and T-2 toxin in maize- and cereal-based feed samples within 20 min.

## 1. Introduction

Mycotoxins are toxic secondary metabolites produced by fungi mainly in *Aspergillus*, *Fusarium* and *Penicillium* genera [1]. These compounds are hazard toxins towards humans and animals, causing harm after ingestion of contaminated food and feeds [2]. Mycotoxins contamination may occur in any stage from farm to table, including field cultivation, harvest, processing, storage and consumption [3]. Recently, the co-occurrence of mycotoxins has been increasingly frequent in agro-food. One of the earliest reports on multi-toxin contamination was found in a maize sample in 1998 [4]. Similarly, feed can be easily and simultaneously infected by various mycotoxins because an optimal environment is present for these processes where fungal spores exist, and they include two, three, or possibly more raw materials. The co-occurrence of mycotoxin in food may result in synergistic and additive toxicological effects in humans or animals [5,6]. Thus, a method to determine the presence of multiple mycotoxins is required to monitor their co-contamination.

In this study, aflatoxin B1 (AFB1), zearalenone (ZEN), and T-2 toxins (T-2) were targets to monitor in feed due to their high toxicity [7,8]. Aflatoxin B1 has been classified as a Group 1 human carcinogen by the International Agency for Research on Cancer [9]. AFB1 can cause irreversible retardation in animals, resulting in economic losses in animal husbandry [10]. ZEN is a non-steroidal estrogenic compound and can cause serious disturbances of the reproductive system in terms of abortions and decreased fertility [11], and estrogenic effects in animals and humans [12]. T-2 toxins cause cytotoxic and immunosuppressive harm by inhibiting DNA and RNA synthesis [13]. The co-occurrence of these mycotoxins is frequently found in the monitoring of mycotoxin in food and feeds. Therefore, monitoring multiple mycotoxins in food and feeds is important, and the development of a rapid assay for the on-site determination of mycotoxins would be invaluable to identify point source contamination.

Several analytical methods for mycotoxin determination have been well-developed including high-performance liquid chromatography (HPLC) and high-performance liquid chromatography/tandem mass spectrometry (HPLC-MS/MS) [14,15]. Despite their excellent accuracy and sensitivity, these methods are relatively complex, expensive, and labor- and time-consuming. Thus, these methods are not suitable for on-site mass sample screening. To solve this problem, several immunoassays such as enzyme linked immunosorbent assay (ELISA) [16] and immune fluorescent assay (IFA) [17] have been well developed and commercial available. However, ELISA requires complex sample preparation, labor-intensive and time-consuming operations, such as coating antigen, blocking and termination. The highly sensitive IFA requires special equipment and is a lab-dependent technique. On the other hand, the immunochromatograpic assay (ICA) is ideally suited for on-site determinations, because of its lab-independence, high sensitivity, specificity and low cost. Gold nanoparticles (AuNPs) is a popular labeling material, due to its ease of synthesis, high stability, good mobility in porous membrane and low susceptibility to aggregation [18]. Several studies have reported multiple target determination based on the AuNPs [19,20]. However, the AuNPs-based lateral flow assay has some obvious drawbacks for multiple targets of interest. With the same AuNPs color, the risk of misreading for multiple targets increases in the small scale on ICS, especially when several test lines exist on the strip. In addition, closely-spaced test zones may cause interference between multiple targets. Thirdly, attempts to improve the resolution by increasing the distance between test lines results in increased membrane costs and assay time [21]. To address this issue, we can achieve clear judgment of different mycotoxins by using multi-color probes.

In this paper, we reported an alternative for an on-site simultaneous detection of AFB1, ZEN and T-2 in maize- and cereal-based feed, based on a multi-color visual ICS. After conjugating anti-mycotoxin monoclonal antibodies with diversely-colored nanoparticles, we developed a multi-color ICS. To evaluate its performance, the detection limit and cut-off values were explored, and a comparison to HPLC–MS/MS was conducted. By using a simple visual readout of colors, an on-site determination method was performed for three major mycotoxins. This proposal is supposed to be an efficient, sensitive and rapid detection method.

## 2. Results and Discussion

### 2.1. Design Principle

**Figure 1:**
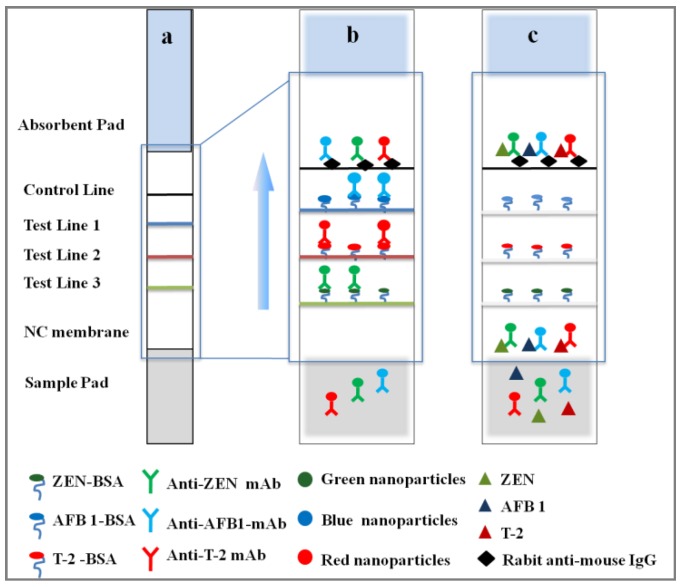
Schematic illustration of: (**a**) multi-color strip; (**b**) for negative samples; and (**c**) for positive samples.

The assay design is shown in Figure 1. The ICS were based on competitive interaction between mycotoxins and their corresponding antigens. When the sample extraction and three monoclonal antibody-nanoparticle (mAb-NPs) conjugates were added onto the sample pad, the mixture flowed toward the NC (nitrocellulose) membrane via capillary action (Figure 1a). In the absence of mycotoxins in the sample extraction, mAb-NPs conjugates were captured by the corresponding antigens pre-immobilized on T lines, resulting in different colored T lines, which is a negative result (Figure 1b). In the presence of mycotoxin in the sample extraction, target of interest competed with the corresponding antigens to react with the corresponding mAb-NPs conjugates. With the increase of mycotoxin content, small amount of free mAb-NPs bound to antigen on T line, causing a shallow coloring of the T line. If the mycotoxins concentration exceeded the cut-off limit, there was no visible test line, which is a positive result, due to the complete binding of the capture reagents (mAb-NPs) by the free analytes (Figure 1c). Regardless of whether the toxin was present in the sample, the control line would be colored due to the interaction between mAb and rabbit anti-mouse immunoglobulin to ensure that the test strip was normal. The absence of a control line would indicate the strip is no longer functional.

### 2.2. ICS Parameters Optimization

**Figure 2:**
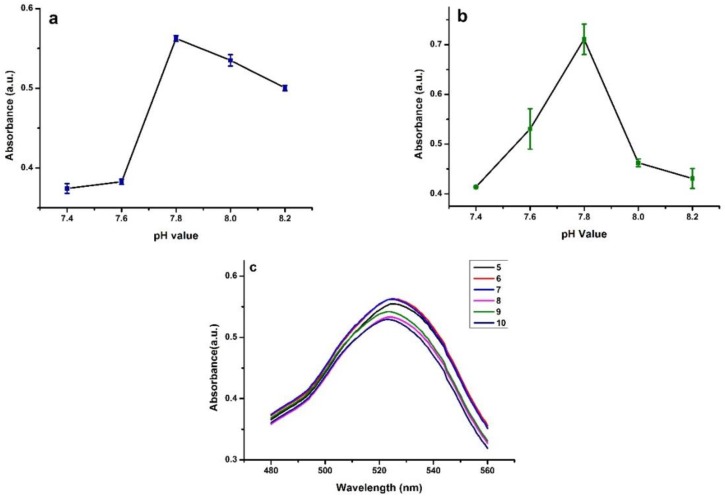
The pH value optimization of: (**a**) mAb-BNPs; (**b**) mAb-GNPs; and (**c**) mAb-AuNPs.

The pH value played a vital role in the process due to its effect on antibody activity and on the interaction between nanoparticles and monoclonal antibodies. Herein, we optimized the pH value and antibody amount in the preparation of these three mAb-NPs conjugates. Commercial carboxyl-modified blue nanoparticles (BNPs) (100 μL) were dissolved in 900 μL boric acid solutions with different pH values (7.2, 7.4, 7.6, 7.8, 8.0 and 8.2). Then, same amount of mAb was added to each tube, respectively. After a 30 min incubation at room temperature, the lower absorbance of supernatant was detected at 595 nm using coomassie blue protein assay kit. The lower absorbance of the supernatant means less antibodies remained, that is, more antibodies were immobilized on the blue nanoparticles. The pH optimization of mAb-modified green nanoparticles (mAb-GNPs) followed the same procedure as for mAb-modified blue nanoparticles (mAb-BNPs). Results showed that 7.4 was the optimal pH values for mAb-BNPs (Figure 2a) and mAb-GNPs (Figure 2b) conjugates. mAb-modified gold nanoparticles (mAb-AuNPs) was optimized for the pH value by titrating the solution using a gradient concentration of K_2_CO_3_ solution (0.1 M) [22]. K_2_CO_3_ solution (1–10 μL) was added to respective 1.5 mL tube with 1 mL synthesized AuNPs. Then, the same amount of mAb was added to each tube. The diameter of the mAb-AuNPs conjugates would increase due to the antibodies immobilization, resulting in a red-shift wavelength of the highest absorbance. The optimized pH value was set as 524 nm at the peak of the max absorption (Figure 2c), where 6 μL of 0.1 M K_2_CO_3_ was added to the solution.

The concentrations of each mAb were optimized to obtain the required visibility and the best sensitivity. The optimal concentrations for each mAb-NPs conjugate were 7, 15 and 20 µg/mL for anti-T-2, anti-AFB1 and anti-ZEN, respectively.

Next, we investigated the influence of the immunoreagent concentration and the type of NC membrane. In an ideal situation under optimal conditions, the concentration of immunoreagent would be minimized to allow considerable sensitivity and color intensity. The optimal immunoreagent concentration was studied by using a checkerboard titration. The optimal amount of AFB1-BSA on the T1 was 0.5 mg/mL, of T-2-BSA was 0.4 mg/mL, of ZEN-BSA was 0.75 mg/mL and rabbit anti-mouse IgG antibody was 0.25 mg/mL. Under these optimal conditions, the multi-component strip indicated good sensitivity and clear test lines.

For membranes with different pore sizes, the HF 135 NC membrane performed better than 90 or 180 membrane types. With a faster chromatography rate, the 90 membrane has a rapid flow rate, causing incomplete immunoreaction and lower sensitivity. This is because the mAb-NPs lack sufficient time to react with the antigen coated on the test line. Conversely, the 180 membrane had sufficient time for reaction with a low chromatography rate, extending the determination time. The immunoreagents could be blocked from leaving the sample pad due to a smaller pore radius.

### 2.3. ICS Performance Evaluation

**Figure 3:**
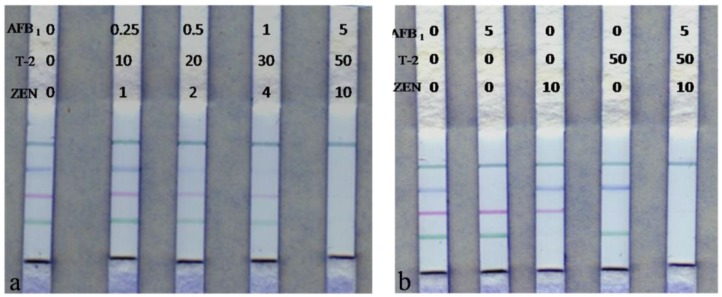
Sensitivity (**a**); and specificity (**b**) of the multicolor immunochromatographic assay.

The visible limit of detection (vLOD) was defined as the minimum analyte concentration where a dramatic decrease in the visibility of the test line was observed. The cut-off level was defined as the lowest concentration at which the test line was completely invisible. The vLOD of the test strip for AFB1, T-2 and ZEN were 0.5 ng/mL, 30 ng/mL and 2 ng/mL, respectively. The cut-off concentrations of ICS were 1, 50 and 10 ng/mL for AFB1, T-2 and ZEN, respectively (Figure 3a). Compared with other reports, these results are better than other strip assays for the detection of AFB1 [18], ZEN [20], and T-2 [23].

To ensure no interference in the simultaneous detection of multiple mycotoxins, we tested sample mixtures containing all three toxins. The three antibodies presented good specificity for their corresponding antigens and the presence of each of the toxins did not affect the signals for the other toxins (Figure 3b). This result is due to the high specificity of these mAbs. Therefore, these multi-colored strips have high specificity and could be applied to the on-site simultaneous determination of multiple targets.

To evaluate the accuracy, five tests were conducted using spiked blank maize and feed samples. Even at low concentrations of multiple mycotoxins, the visible concentration and the cut-off values were consistent between the repeats (Table 1). Thus, this proposal exhibited good accuracy.

### 2.4. Comparison between ICS and HPLC-MS/MS

A comparison between the multi-color strip and HPLC-MS/MS was performed using six maize and feed samples. All samples were analyzed with both ICS and HPLC-MS/MS. The mass spectrum conditions are listed in Table S1. Briefly, 1 mL sample extraction was diluted with 4 mL diluent in a vial and incubated with mAb-NPs conjugates for 10 min. This incubation ensured a sufficient reaction between the mAb-NPs conjugates and the mycotoxins. The incubation solution was then loaded on ICS and reacted for 10 min. Afterwards, an identical sample extract filtered through a 0.22 μm pore membrane was used for HPLC-MS/MS analysis. As shown in Figure 2a, the color intensity of the T lines decreased as the mycotoxin concentrations increased. Furthermore, no T line was observed when the concentrations of AFB1, ZEN and T-2 were above the cut-off value. The results of the multi-color strip were in good agreement with those from the instrumental results (Table 2).

## 3. Conclusions

In this paper, by utilizing multi-color visual immunochromatography, we propose an on-site simultaneous semi-quantification of AFB1, T-2 toxin, and ZEN in maize and feed samples. This proposal addresses technical issues in the determination of co-occurrence of multiple mycotoxins contamination. We combine colored nanoparticles and specific antibodies to increase the visibility on the multicolor strip. The sensitivity of the ICS is found to be better than, or similar to, the AuNPs-based immunochromatographic strip. Using this rapid and reliable method, the results could be observed with the naked eye within 20 min. The visible detection limit and cut-off value of the proposed assay are 0.5, 30, and 2, and 1, 50, and 10 ng/mL, for AFB1, T-2 and ZEN, respectively. More importantly, distinguishing different mycotoxins of interest with different colors is easier using the developed ICS with the naked eye. The immunochromatographic strip could be extensively applied in the on-site screening of other contaminants by simply changing the monoclonal antibodies.

## 4. Materials and Methods

### 4.1. Reagents and Materials

*N*-hydroxyl-succinimide (NHS), hydrogen tetrachloroaurate (III) hydrate, 1-(3-(dimethyl amino)propyl)-3-ethylcarbodiimide hydrochloride (EDC), chemical standard of AFB1, ZEN and T-2, BSA (bovine serum albumin), AFB1-BSA conjugate, T-2-BSA conjugate and rabbit anti-mouse immunoglobulin (IgG) were purchased from Sigma-Aldrich (St. Louis, MO, USA). ZEN-BSA conjugate was purchased from Disy Bio-Tech Co., Ltd. (Beijing, China). Blue and green nanoparticles purchased from Merck (Darmstadt, Germany). Acetonitrile (ACN), methanol and formic acid was purchased from SinopharmChemical Reagent Co., Ltd. (Shanghai, China). Coomassie blue plus protein assay kit was purchased from Thermo Fisher Scientific Inc. (Waltham, MA, USA) Water was obtained from a Milli-Q purification system (Millipore, Bedford, CA, USA). All other inorganic chemicals and solvents were of analytical reagent grade. Sample diluents were 1% (*m*/*v*) BSA, 1% sucrose, 1% polyvinylpyrrolidone and 2.5% Tween-20 made in ultrapure water. Nitrocellulose (NC) membranes, sample pads and absorbent pads were purchased from Millipore Corporation (Bedford, MA, USA). Different NC pore sizes were tested using Millipore Hi-Flow Plus HF090, 135 and 180(Millipore, Bedford, MA, USA). Maize- and cereal-based feed were from local market. Samples with undetected mycotoxins (AFB1, ZEN and T-2) after LC-MS/MS analysis was selected as blank samples and used in spiking and recovery experiments.

### 4.2. Instrumentation

A commercial mill was purchased from Joyoung Co., Ltd. (Wuhan, China) for sample pretreatment. The XYZ3050 Dispensing Platform, LM4000 Batch Laminator and CM4000 Guillotine Cutter from BioDot (Irvine, CA, USA) were used to manufacture ICS. The microwave was purchased from Midea Co., Ltd. (Wuhan, China) for AuNPs synthesis. The high-speed freezing centrifuge (CF16RX) was from Hitachi Koki Co., Ltd. (Tokyo, Japan). The high-performance liquid chromatography system/tandem mass spectrometry (HPLC-MS/MS) was from Agilent Tech (Santa Clara, CA, USA).

### 4.3. AuNPs Synthesis

Two milliliters of 1% HAuCl_4_ and 200 mL H_2_O were refluxed for 5 min by microwave. Then, 5.2 mL 1% (*w*/*v*) filtered trisodium citrate were added to the boiling mixture quickly, and was refluxed for another 4 min. After cooling to room temperature, the solution was transferred to a 200 mL volumetric flask and diluted with ultrapure water to volume and mixed. The AuNPs were stored at 4 °C before use.

### 4.4. Monoclonal Antibodies (mAb) Production

Anti-AFB1 monoclonal antibody was prepared in mouse ascites fluid according to previously reported methods. In brief, antigen was emulsified with immune-adjuvant and inoculating into BALB/c mice. After four immunizations, hybridomas were obtained using cell fusion and a two-step ELISA screening procedure was used to identify the hybridomas with the highest sensitivity. Hybridomas from cell cultures were then used to inoculate immune-adjuvant-treated BALB/c mice to obtain ascites fluid. We identified several monoclonal antibodies that retained high sensitivity and specificity through purification, dialysis, freeze drying and characterization. Monoclonal antibodies against ZEN and T-2 were produced via the similar method. All experiments were approved by the Laboratory Animal Monitoring Committee of Hubei Province (No. 42000600015662; Date of Approval: 5 July 2016).

### 4.5. mAb-Nanoparticles (mAb-NPs) Conjugation

#### 4.5.1. Preparation of mAb-BNPs (Blue Nanoparticles) for AFB1 Detection

One milliliter of pH-adjusted BNPs was activated by an incubation with 50 μ LEDC (20 mg/mL) and 50 μL NHS (15 mg/mL). Then, 20 μL L-cysteine (Sigma-Aldrich, St. Louis, MO, USA) (50 mg/mL) were added to ensure sufficient reaction between BNPs and anti-AFB1 monoclonal antibody. After stirring for 2 h, the mixture was centrifuged at 13,800× *g* at 4 °C for 15 min and the pellet was re-suspended in borate buffer (2 mM pH 7.4). The optimized amount of anti-AFB1 mAb was added dropwise and stirred for 4 h. A total of 100 μL of 1% filtered BSA was added to block the excess active sites on the surface of the BNPs. Afterwards, the solution was centrifuged at 13,800× *g* at 4 °C for 15 min and the final conjugate was re-suspended in 100 μL borate buffer (2 mM pH 7.4) and stored at 4 °C before use.

#### 4.5.2. Preparation of mAb-AuNPs Conjugates for T-2 Toxin Detection

mAb-AuNPs conjugates for T-2 toxin were prepared as follows. The optimized pH values and optimum antibody amounts were identified as previous reports with minor modification [3]. The synthesized AuNPs solutions (3 mL) were adjusted to the optimal pH with 0.1 M K_2_CO_3_ (15 µL), and then 300 μL of anti-T-2 mAb (0.1 mg/mL) were added dropwise. After a 30 min gentle stirring at room temperature, 368 μL of 10% (*w*/*v*) filtered BSA were added dropwise to block the excess epitopes on AuNPs surface. After a 30 min incubation, the mixture was centrifuged at 800× *g* at 4 °C for 15 min to precipitate the aggregated particles, and then suspension was centrifuged at 13,800× *g* for 30 min. Afterwards, antibody-modified nanoparticles were re-suspended in 300 μL borate buffer (2 mM pH 7.4) and stored at 4 °C before use.

#### 4.5.3. Preparation of mAb-GNPs (Green Nanoparticles) for ZEN Detection

The mAb-GNPs conjugates for ZEN detection were prepared using similar procedure as for AFB1 with minor modifications. Briefly, after activation by EDC/NHS at the optimal pH value, GNPs reacted with anti-ZEN-mAb by gentle stirring. Then, the conjugate was suspended in 100 μL borate buffer (2 mM pH 7.4) and stored at 4 °C after the processes of centrifugation, re-suspension and BSA-blocking.

### 4.6. Preparation of ICS

#### 4.6.1. Immobilization of Antigens

The three antigens of AFB1, T-2 and ZEN were coated on NC membranes successively as three test lines, T1 line, T2 line, and T3 line (Figure 2), and the rabbit anti-mouse IgG was coated as the control line (C line) using a BioDot XYZ platform (Beijing New Point biotechnology company Litd., Beijing, China) at jetting rates of 0.5 μL/cm for T2 and 0.6 μL/cm for T1, T3 and C line. The coated NC membrane was dried for 2 h at 37 °C and sample pad were treated with blocking buffer (0.01 mol/L pH 7.4 PBS + 2% BSA + 2.5% Sucrose + 0.02% NaN_3_) and dried overnight at 37 °C. Afterwards, the antigen-coated NC membranes were assembled with absorbent pads and sample pads onto the backing card.

#### 4.6.2. ICS Assembly

The antigen-coated NC membrane was affixed in the middle of an adhesive backing card. The absorbent pads are stamped sequentially next to the NC membrane with a 2 mm overlap at the NC membrane end. The sample pad was pasted next to the NC membrane with 2 mm overlap. Finally, the whole assembled back plate was cut into 4 mm width strips.

### 4.7. Sample Preparation

Twenty-five grams of maize and feed samples were ground to fine powder by treating in the grinding machine for 2 min respectively. Five grams of the resulting powder were mixed with 20 mL 70% (*v*/*v*) methanol/water and vigorously stirred for 20 min. The mixture was then centrifuged at 3500× *g* at 4 °C for 5 min. Afterwards, 1 mL supernatant was diluted in 4 mL sample diluents prior to further ICS analysis. For HPLC-MS/MS analysis, the identical sample extraction described above should be filtered through a 0.22 μm pore membrane.

### 4.8. Sensitivity, Specificity and Accuracy Evaluation of the ICS

The sensitivity of the strips was evaluated using samples extract containing a series of concentrations of the three analytes (AFB1/T-2/ZEN 0/0/0, 0.25/10/1, 0.5/20/2, 1/30/4, and 5/50/10 ng/mL). These sample extract were analyzed by ICS to determine the visible limit of detection and cut-off level of different analytes presenting no color at the test line. The spiked maize and feed sample extracts with different concentrations of AFB1/T-2/ZEN (0/0/0, 5/0/0, 0/0/10, 0/50/0, and 5/50/10 ng/mL) were used to validate the specificity of ICS. A 150-μL mixture of spiked extract and mAb-NPs capture probes was incubated for 10 min at 37 °C and then subjected to ICS. After 10 min incubation, the visible results were judged by the naked eyes. To determine the accuracy, samples containing multi-toxins were analyzed and repeated 5 times.

### 4.9. Validation via HPLC-MS/MS Analysis

To validate the application of the ICS in a real sample, the diluent sample extract was used for HPLC-MS analysis. The chromatographic column was a C18 (100 mm × 2 mm, 3 μm particle size) with a 10 mL injection volume. The mobile phase was acetonitrile (ACN) and 0.1% formic acid at a flow rate of 200 μL/min. The test compounds were eluted using the following gradient conditions: 0–8.5 min, 15–50% ACN; 8.5–10 min, 50% ACN; 10–11.5 min, 50–70% ACN; 11.5–13.5 min, 70% ACN; 13.5–15 min, 70–100% ACN; 15–18 min, 100–50% ACN; 18–25 min, 50–15% ACN.

## Figures and Tables

**Table 1:** Results of accuracy of ICS.

AFB1/T-2/ZEN (ng/mL)	ICS Visual Result (*n* = 5)			
	Test Line 1	Test Line 2	Test Line 3	Control Line
0/0/0	−	−	−	−
0.25/10/1	−	−	−	−
0.5/20/2	+	−	+	−
1/30/4	++	+	+	−
5/50/10	++	++	++	−

−: Negative result; +: Positive result, T line was observed; ++: Positive result, T line was not observed.

**Table 2:** Comparison of the analysis results of AFB1, ZEN and T-2 toxin in maize and feedstuff by developed ICS and LC-MS/MS.

No.	Sample	ICA (*n* = 4)												Result of LC-MS/MS (ng/mL)		
		AFB1				T-2				ZEN				Found ± SD (ng/mL)		
		1	2	3	4	1	2	3	4	1	2	3	4	AFB1	T-2	ZEN
1	maize	−	−	+	+	−	−	−	−	++	++	++	++	0.55 ± 0.07	1.73 ± 0.46	45.72 ± 9.11
2	maize	+	+	++	++	+	+	+	++	−	−	−	−	1.33 ± 0.35	47.67 ± 6.40	ND
3	maize	++	++	++	++	++	++	++	++	−	−	−	−	1.62 ± 0.45	76.02 ± 7.19	ND
4	feedstuff	−	−	−	−	−	−	−	−	+	+	+	+	ND	16.86 ± 0.73	8.53 ± 0.46
5	feedstuff	−	−	−	−	−	−	−	−	++	++	++	++	ND	ND	18.63 ± 1.81
6	feedstuff	++	++	++	++	−	−	−	−	++	++	++	++	2.56 ± 0.32	11.07 ± 0.99	11.87 ± 0.47

−: Negative result, AFB1 < 0.5 ng/mL, T-2 < 30 ng/mL, ZEN < 2 ng/mL. T line was obvious; +: Positive result, 0.5 ng/mL ≤ AFB1 ≤ 1 ng/mL, 30 ng/mL ≤ T-2 ≤ 50 ng/mL,2 ng/mL ≤ ZEN ≤ 10 ng/mL. T line was observed; ++: Positive result. AFB1 ≥ 1 ng/mL, T-2 ≥ 50 ng/mL, ZEN ≥ 10 ng/mL, T line was not observed; ND: Not detected.

## Supplementary Materials

The following are available online at http://www.mdpi.com/2072-6651/10/2/87/s1, Table S1: Mass spectrum conditions of LC-MS/MS analysis.
