# Supplementary material for: An On-Site Simultaneous Semi-Quantification of Aflatoxin B1, Zearalenone, and T-2 Toxin in Maize- and Cereal-Based Feed via Multicolor Immunochromatographic Assay
Source: Toxins (Basel). 2018 Feb 17;10(2):87. doi: 10.3390/toxins10020087 (PMC5848188; doi:10.3390/toxins10020087)
Supplement: Supplementary file 1 [file toxins-10-00087-s001.pdf]

SupplementaryMaterials: An On-Site Simultaneous Semi-quantification of Aflatoxin B1, Zearalenone, and T-2 Toxin in Maize- and Cereal-based Feed via Multicolor Immunochromatographic Assay

Lin Xu, Zhaowei Zhang, Qi Zhang, Wen Zhang, Li Yu, Du Wang, Hui Li and Peiwu Li

Table S1. Mass spectrum conditions of LC-MS/MSanalysis.

Compound	Mode	Precursor ion	Product ion(m/z)	Collision energy (eV)
AFB1	ESI^+	$313[M+H]^+$	285.0	35
			241.1	35
ZEN	ESI^-	$317[M-H]^-$	130.8	30
			175.9	25
T-2	ESI^+	$489[M+H]^+$	245.2	26
			387.3	20
